# Benign, persistent, and invasive: mechanistic and translational approaches to middle‑ear cholesteatoma

**DOI:** 10.37349/etat.2026.1002359

**Published:** 2026-02-24

**Authors:** Pinelopi Samara, Michail Athanasopoulos, Ioannis Athanasopoulos

**Affiliations:** N.N. Petrov Research Institute of Oncology, Russian Federation; ^1^Children’s Oncology Hospital “Marianna V. Vardinoyannis-ELPIDA”, 11527 Athens, Greece; ^2^Otolaryngology-Head and Neck Surgery, Athens Pediatric Center, 15125 Athens, Greece

**Keywords:** cholesteatoma, middle ear, recurrence, oxidative stress, inflammation, bone erosion, epigenetics, microbiome

## Abstract

Acquired middle-ear cholesteatoma is a histologically benign keratinizing squamous epithelial lesion that paradoxically exhibits locally destructive, recurrent, and invasive behavior, often resulting in ossicular erosion, hearing loss, labyrinthine fistula, and, rarely, intracranial complications. Surgical excision remains the primary management strategy; however, recurrence is common due to persistent microenvironmental drivers. Recent mechanistic studies—including single-cell transcriptomics, spatial proteomics, and epigenetic profiling—reveal a multifactorial pathogenesis orchestrated by chronic inflammation, proteolytic extracellular-matrix remodeling, osteoclast activation via RANKL and activin A, epithelial plasticity with partial epithelial-to-mesenchymal transition (EMT), and a dysbiotic, biofilm-forming microbiome. Emerging evidence further implicates oxidative stress, RNA and epigenetic modifications, miRNA dysregulation, and immune cell infiltration as central modulators of lesion chronicity and bone resorption. Collectively, these processes establish a self-sustaining pro-osteolytic microenvironment that drives bone erosion and postoperative recurrence. Cholesteatoma recapitulates several features of malignant lesions—hyperproliferation, local invasion, and stromal/immune cell recruitment—yet remains fundamentally benign, lacking metastatic potential and genomic instability. Its aggression is ecological rather than genetic, highlighting the potential for microenvironment-directed, precision-based strategies. Adjunctive approaches may include local delivery of modulatory agents, targeted interference with inflammatory, proteolytic, osteoclastogenic, and microbial axes, and biomarker-guided patient stratification. Preclinical and early-phase experimental studies assessing target engagement, radiologic stabilization, and molecular surrogates of efficacy could inform safer, mechanism-driven interventions that complement surgery, reduce recurrence, and preserve hearing. Integrating molecular pathobiology with clinical strategy positions cholesteatoma as a model for benign yet locally aggressive, microenvironment-driven disease, providing a roadmap for translational therapies with direct relevance to surgical practice.

## Introduction

Acquired middle-ear cholesteatoma is a locally destructive lesion of the tympanic cavity, composed of keratinizing squamous epithelium. Clinically, it presents with progressive conductive hearing loss, ossicular erosion, and, in severe cases, labyrinthine fistula, facial nerve palsy, or intracranial complications [1, 2]. Despite its histologically benign nature, cholesteatoma is notorious for aggressive local destruction and high recurrence rates after incomplete excision [3], making it a condition of significant surgical and clinical concern.

The term cholesteatoma, derived from the Greek “chole” (cholesterol), “steat” (fat), and “oma” (tumor), is historically misleading. When Johannes Müller first described the lesion, he interpreted it as a fatty tumor, a characterization later proven inaccurate, as these lesions contain neither cholesterol nor adipose tissue [1]. Cholesteatomas are broadly classified into congenital and acquired forms. Congenital lesions arise behind an intact tympanic membrane, typically diagnosed in early childhood. Acquired cholesteatomas develop secondary to chronic otitis media or Eustachian tube dysfunction and are further subclassified as primary—arising from retraction pockets without prior trauma—or secondary, resulting from tympanic membrane perforation or prior surgery. Anatomical origin also influences behavior: pars flaccida (attic) and pars tensa (posterior quadrant) lesions exhibit distinct growth patterns and differential risks for ossicular erosion [4]. Epidemiologically, cholesteatoma affects approximately 3 per 100,000 children and 9 per 100,000 adults annually, with a slight male predominance, peaking in the second to fourth decades for acquired forms [5].

At the molecular level, cholesteatoma pathogenesis is increasingly understood as microenvironment-dependent, with persistent inflammation serving as a central driver. Chronic cytokine activity—including interleukin (IL)-6, tumor necrosis factor alpha (TNF-α), IL-1β, and IL-8—and stress-response proteins such as heat shock protein 27 (HSP27) promote keratinocyte proliferation, immune cell recruitment, and tissue remodeling [6]. Concurrently, matrix degradation mediated by matrix metalloproteinase (MMP)-2 and MMP-9, along with osteoclast activation, underlies the characteristic bone erosion observed clinically [7]. These processes are further amplified by oxidative stress and dysregulation of miRNAs and epigenetic modifications, which modulate apoptosis, proliferation, and inflammatory signaling. Partial epithelial-to-mesenchymal transition (EMT) contributes to keratinocyte motility and stromal remodeling, recapitulating certain hallmarks of neoplastic invasion without conferring metastatic potential [8].

Surgical excision—ranging from canal-wall-up to canal-wall-down tympanomastoidectomy—remains the cornerstone of management, aiming to eradicate disease and prevent recurrence [9]. However, these mechanistic insights provide opportunities to identify prognostic biomarkers, guide surveillance, and develop adjunctive therapies targeting inflammation, matrix remodeling, or oxidative stress.

The mini-review synthesizes emerging molecular and cellular knowledge into acquired middle-ear cholesteatoma with a translational lens, highlighting the microenvironmental drivers of local tissue destruction, recurrence, and osteolysis. By connecting mechanistic understanding to precision, locally delivered adjunctive strategies and biomarker-guided approaches, it provides a novel framework to complement surgical management. This perspective positions cholesteatoma as a model for benign yet locally aggressive, microenvironment-driven disease and offers actionable directions for future translational and clinical research.

## Core pathobiological axes and candidate molecular targets

**Figure 1:**
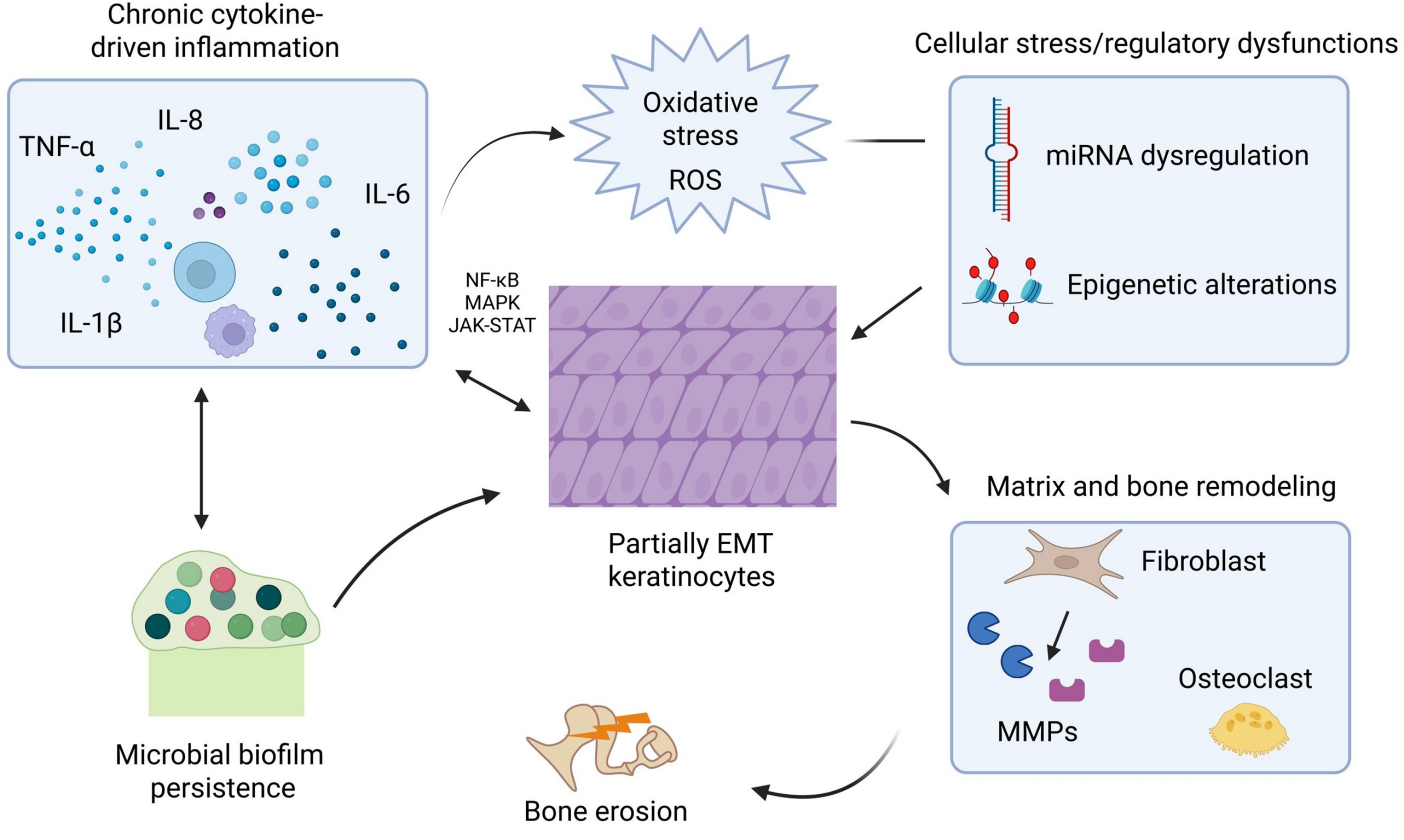
**Illustration depicting cytokine-induced inflammatory cascades and microenvironmental interactions in cholesteatoma.** Chronic cytokine signaling activates NF-κB, MAPK, and JAK–STAT pathways in keratinocytes, promoting hyperproliferation and partial EMT. Oxidative stress and miRNA/epigenetic dysregulation reinforce these epithelial changes. Crosstalk among fibroblasts, immune cells, and osteoclasts drives proteolytic matrix remodeling and bone erosion. Persistent microbial biofilms sustain inflammation and oxidative stress, creating a self-perpetuating microenvironment that underpins cholesteatoma progression and recurrence. “Illustration depicting cytokine-induced inflammatory cascades and microenvironmental interactions in cholesteatoma” created in BioRender. Sam, P. (https://BioRender.com/c605dyv) is licensed under CC BY 4.0.

Chronic cytokine-driven inflammation activates nuclear factor kappa B (NF‑κB), mitogen-activated protein kinase (MAPK), and janus kinase/signal transducer and activator of transcription (JAK–STAT) signaling, which together with oxidative stress and miRNA/epigenetic dysregulation drives keratinocyte proliferation and partial EMT. These epithelial changes, in concert with proteolytic matrix remodeling, osteoclast activation and fibroblast–immune cross-talk, result in bone erosion, while persistent microbial biofilms amplify local inflammation, creating a self-sustaining microenvironment that underpins cholesteatoma growth and recurrence (Figure 1, Table 1) [6, 8].

**Table 1:** Key molecular and cellular mechanisms underlying cholesteatoma pathogenesis.

**Pathobiological axis**	**Representative molecules/Pathways**	**Functional outcome/pathophysiological role**	**Key references**
Chronic inflammation and immune signaling	NF-κB, MAPK, JAK–STAT, TLR4, TNF-α, IL-1β, IL-6, IL-8	Sustains cytokine-driven inflammation, enhances keratinocyte proliferation, and promotes osteoclastogenic signaling	[6, 10, 12]
Proteolytic matrix remodeling	MMP-2, MMP-8, MMP-9, MMP-14, cathepsins	Degrades extracellular matrix and bone tissue, facilitating epithelial invasion and ossicular erosion	[7, 13, 14, 33]
Osteoclast-driven bone resorption	RANKL, OPG, activin A, PTHrP	Promotes osteoclast differentiation and activity, driving localized bone destruction	[11, 15]
Epithelial plasticity and partial EMT	OPN–CD44–AKT, ZEB2	Enables keratinocyte survival, migration, and matrix interaction	[8, 16]
Microbiome and biofilm persistence	Microbial biofilms, TLR activation, quorum-sensing, dysbiosis	Maintains chronic inflammation via persistent innate immune activation and antimicrobial resistance	[17–19]
Oxidative stress	ROS, MPO	Amplifies inflammatory signaling, protease induction, and DNA/RNA oxidative damage	[8, 20]
Epigenetic and RNA modifications	DNA methylation, histone acetylation (H3K27ac), m6A-modified circular RNAs	Alters gene expression programs governing proliferation, inflammation, and ECM remodeling	[21, 22]
miRNA dysregulation	miR-223-3p, miR-142-5p, miR-21-5p, exosomal miRNAs	Modulates inflammatory signaling, osteoclastogenesis, and intercellular communication; potential biomarkers	[23–25]
Immune cell infiltration and phenotypes	M1/M2 macrophage polarization (CD80^+^/CD163^+^), T-cell subsets, mast cells	Shapes inflammatory milieu, influences matrix degradation, and contributes to bone erosion and fibrosis	[26, 27]

OPG: osteoprotegerin; PTHrP: parathyroid hormone-related protein; OPN: osteopontin; MPO: myeloperoxidase; m6A: N6-methyladenosine; ZEB2: zinc finger E-box binding homeobox 2.

### Chronic inflammation and immune signaling

Persistent activation of innate immune pathways is a central driver of cholesteatoma. Cytokines such as TNF‑α, IL‑1β, IL‑6, and IL‑8 converge on NF‑κB, MAPK, and JAK–STAT hubs, promoting keratinocyte proliferation, suppressing apoptosis, and inducing protease expression [10]. Single-cell analyses reveal complex crosstalk among keratinocytes, macrophages, and fibroblasts that sustains local inflammation and osteoclastogenic signaling [11]. The composition and activation states of immune infiltrates—macrophage polarization, neutrophil activity, and T-cell subsets—modulate both inflammatory tone and bone resorption [12], positioning immune profiling as a promising biomarker and therapeutic strategy. These inflammatory pathways are among the most consistently validated mechanisms in cholesteatoma pathogenesis, supported by immunohistochemical, transcriptomic, and functional studies.

### Proteolytic matrix remodeling and epithelial invasion

Cholesteatoma exhibits a protease-rich microenvironment that drives local tissue destruction. Elevated levels of MMPs—particularly MMP-2, MMP-8, MMP-9, and MMP-14—along with cathepsins, contribute to degradation of basement membranes and bone matrix, facilitating epithelial invasion and ossicular erosion. Recent studies confirm that MMP-2 and MMP-9 expression correlates with the severity of bone resorption and recurrence risk [13], while MMP-14 has emerged as a key regulator of osteolytic activity and fibroblast-epithelial cross-talk [14].

### Osteoclast-driven bone resorption and stromal cross-talk

Bone erosion in cholesteatoma is an active, osteoclast-mediated process. Stromal fibroblasts and inflammatory cells secrete receptor activator of nuclear factor kappa-B ligand (RANKL), activin A, and parathyroid hormone-related protein (PTHrP), tipping the RANKL/osteoprotegerin (OPG) balance toward osteoclastogenesis. Single-cell profiling has identified an activin A–high fibroblast subset that links stromal inflammation to osteoclast recruitment, suggesting potential targets for local anti-RANKL or anti-activin strategies. RANKL expression and activin A signaling—observed in osteoclastogenic fibroblasts within cholesteatoma tissue—may contribute to increased osteoclastogenesis and consequent bone resorption, possibly reflecting more pronounced local tissue destruction in some lesions [11, 15].

### Epithelial plasticity and partial EMT

Keratinocytes within cholesteatoma adopt features of partial EMT, including downregulation of junctional proteins, upregulation of motility and mesenchymal regulators such as zinc finger E-box binding homeobox 2 (ZEB2), and activation of osteopontin (OPN)–CD44–AKT signaling that supports survival and matrix interaction [16]. This hybrid epithelial-mesenchymal state facilitates local expansion and invasion without malignant transformation.

### Microbiome and biofilms

Microbial biofilms persist within cholesteatoma, sustaining chronic activation of toll‑like receptors (TLRs) and NF‑κB signaling. The extracellular matrix of these biofilms protects microbes from host immunity and limits antibiotic penetration [17]. Recent sequencing studies show that cholesteatoma harbors a distinct bacterial community dominated by biofilm‑forming taxa [18]. Anti‑biofilm strategies—including DNases, quorum-sensing inhibitors, or sequencing-guided antimicrobials delivered via mucoadhesive carriers—may disrupt microbial drivers of chronic inflammation and complement surgical and pharmacologic therapy [19].

### Oxidative stress

Reactive oxygen species (ROS) and oxidative stress markers are elevated in cholesteatoma, including lipid peroxidation products, altered antioxidant defenses, and increased myeloperoxidase (MPO) [8]. Oxidative stress amplifies inflammation, promotes matrix degradation, and may drive keratinocyte proliferation and DNA/RNA modifications. Targeting ROS—systemically or via local antioxidant depots—could represent a plausible adjunctive strategy to mitigate downstream effects such as protease induction and osteoclast activation in cholesteatoma [20].

### Epigenetic alterations and RNA modifications

Beyond classical genetic changes, emerging evidence indicates that cholesteatoma progression is influenced by epigenetic and RNA-level modifications. DNA methylation, histone modifications, and RNA modifications—particularly N6-methyladenosine (m6A)—regulate keratinocyte proliferation, inflammatory responses, and extracellular matrix remodeling [8]. For example, acetylation of histone H3 at lysine 27 (H3K27ac), linked to super-enhancer activity at loci such as forkhead box protein C2 (FOXC2), has been observed in human cholesteatoma tissue, highlighting aberrant chromatin activation driving cellular proliferation and tissue remodeling [21]. Altered m6A patterns in circular RNAs and distinctive DNA methylation signatures further point to potential biomarkers and therapeutic targets, including inhibitors of specific epigenetic writers/readers or modulators of RNA modification pathways [22]. While these regulatory mechanisms are supported by recent high-resolution molecular studies, their functional hierarchy and therapeutic tractability remain to be fully elucidated.

### miRNA dysregulation

Small non‑coding RNAs—including miRNAs and exosomal miRNAs—are consistently dysregulated in cholesteatoma, and these alterations impact key processes such as inflammatory signaling, epithelial proliferation, and osteoclastogenesis [23]. Recent studies have identified numerous miRNAs that are consistently up- or down-regulated in cholesteatoma tissue, including miR‑223‑3p, miR‑142‑5p, and miR‑21‑5p, which influence cytokine production and bone remodeling [24]. Exosomal miRNAs appear to mediate communication between epithelial cells, stromal fibroblasts, and immune cells [25], suggesting their potential both as biomarkers and as therapeutic targets. Strategies such as locally delivered miRNA mimics or inhibitors could be used to correct these dysregulations and modulate disease activity.

### Immune cell infiltration and phenotypes

Building on the immune signaling pathways described previously, this section examines the immune cell populations, their polarization, and spatial phenotypes that mediate cytokine-driven tissue remodeling and bone erosion in cholesteatoma. The composition, distribution, and activation states of immune cells critically influence cholesteatoma pathophysiology. Macrophage polarization toward the pro-inflammatory M1 phenotype (CD80^+^) has been associated with increased local cytokine expression, MMP activity, and greater ossicular and temporal bone erosion. In contrast, M2 macrophages (CD163^+^) are linked to tissue remodeling and fibrosis, reflecting a dynamic and heterogeneous inflammatory environment [26]. Mast cells have also been implicated in cholesteatoma formation and bone erosion, suggesting broader participation of innate immune effectors [27]. Further immunophenotypic studies are needed to clarify the contributions of T-cell subsets and macrophage–stromal interactions, which may inform future immunomodulatory approaches such as local cytokine blockade or macrophage-targeted therapy.

## How cholesteatoma mimics—and diverges from—malignant disease

Cholesteatoma recapitulates several molecular and microenvironmental features commonly observed in neoplasia: focal hyperproliferation, chronic activation of pro-survival and inflammatory pathways (NF-κB, MAPK, JAK–STAT), protease-driven local tissue invasion (MMP family, cathepsins), recruitment of osteoclastogenic signals, and acquisition of a partial EMT phenotype that facilitates local expansion [6]. Concurrent epigenetic and small-RNA dysregulation, oxidative stress, and a tumor-like stromal–immune network further enhance cellular plasticity and matrix remodeling [8], producing a histomorphologic and functional phenotype that can appear “quasi-oncologic”. This “quasi-oncologic” pattern, characterized by invasive growth, stromal activation, and recurrence without metastatic potential, parallels other benign yet locally aggressive conditions such as desmoid-type fibromatosis (aggressive fibromatosis), endometriosis, keloids, and pigmented villonodular synovitis.

Very recent work has provided additional molecular evidence supporting cholesteatoma’s quasi-oncologic behavior. Somatic proto-oncogene mutations in MYC (2 of 22 patients) and NOTCH1 (5 of 22 patients) have been identified in a subset of middle-ear cholesteatomas, correlating with local bone destruction [28]. These findings offer key mechanistic insight, demonstrating that cholesteatoma’s aggressive local expansion is not purely inflammatory but may also involve oncogenic signaling pathways. The mutations are not universal, indicating that only a fraction of lesions—approximately 32% in this cohort—may be genetically predisposed to pronounced bone erosion (selective aggression). While this represents the first molecular evidence linking proto-oncogene alterations to cholesteatoma behavior, the small sample size underscores the need for larger studies to validate and extend these observations.

Importantly, cholesteatoma continues to lack hallmark features of malignant neoplasia, including metastatic potential, widespread chromosomal instability, and autonomous growth independent of local microenvironmental cues [29]. Apoptotic and cell-cycle checkpoints remain largely intact, and proliferation is spatially constrained to the lesion microenvironment. Conceptually, these findings reinforce the view of cholesteatoma as a benign pathology in which oncogenic programs are co-opted by a chronic inflammatory ecosystem, producing aggressive local behavior without true neoplastic transformation. This distinction has practical implications: therapeutic strategies should focus on the lesion’s microenvironmental dependencies—such as inflammation, proteolysis, osteoclast recruitment, biofilm persistence, redox imbalance, and epigenetic/RNA dysregulation [10]—rather than relying on systemic cytotoxic regimens designed for malignant disease.

## Translational pathways and research priorities

Cholesteatoma’s locally aggressive, microenvironment-dependent behavior makes it particularly suited for precision, locally delivered adjuncts that complement surgical excision. Intervention may be approached across three temporal windows [30, 31]:

1) Preoperative stabilization: Short courses of topical or local agents aimed at suppressing inflammation, reducing protease activity, and limiting osteoclast-mediated bone loss before surgery.

2) Intraoperative margin therapy: Targeted depots applied to surgical margins containing anti-protease, antioxidant, anti-biofilm, or immune-modulatory agents to neutralize residual microscopic disease and reduce epithelial seeding.

3) Postoperative sustained-release therapy: Depot formulations in the mastoid or epitympanum designed to provide continuous pharmacologic action during the early wound-healing period, potentially lowering recurrence risk.

Preclinical validation could utilize organotypic cholesteatoma–bone co-cultures, *ex vivo* human explants, and selective *in vivo* otic models to replicate epithelial–stromal–osteoclast interactions, biofilm persistence, and microenvironmental regulation, while evaluating local pharmacokinetics, ototoxicity, vestibular safety, and delivery efficiency [32–34]. Current research has largely emphasized surgical technique or imaging, with molecularly targeted adjuncts remaining uncommon. Topical 5‑fluorouracil has been applied in only limited external-canal cases [35], and systematic trials addressing mechanistic pathways are lacking, highlighting an opportunity for biomarker-guided, mechanism-driven early-phase studies.

### Clinical trial design may incorporate

1) Axis-specific interventions such as local inhibition of RANKL or activin A to suppress osteoclastogenesis, MMP inhibitors to limit proteolytic bone erosion, antioxidant or redox-modulating agents to mitigate oxidative stress, epigenetic or RNA-modifying pathway modulators to regulate epithelial plasticity, and anti-biofilm strategies (e.g., DNases or quorum-sensing inhibitors) to disrupt persistent microbial inflammation.

2) Local delivery strategies, including mucoadhesive hydrogels, nanoparticles, or intraoperative depots [30, 31], to enhance efficacy while minimizing systemic exposure.

3) Biomarker-guided cohorts and endpoints using molecular surrogates (gene expression, miRNA/epigenetic signatures), radiologic stabilization of bone, and functional safety measures (audiometry, vestibular testing).

4) Rigorous safety monitoring and extended follow-up to capture potential delayed recurrence.

Cholesteatoma demonstrates substantial clinical and molecular heterogeneity—congenital *versus* acquired forms, pediatric *versus* adult presentations—driven by intersecting inflammatory, proteolytic, osteoclastogenic, microbial, and regulatory (oxidative stress, epigenetic/miRNA) axes [1, 6]. Advancing treatment may focus on:

- Mechanistic characterization: Defining contributions of osteoclast-stromal signaling, partial EMT, biofilm persistence, and oxidative/epigenetic dysregulation to lesion expansion and bone erosion.
- Biomarker discovery and validation: Identifying predictive and pharmacodynamic markers for patient stratification and early therapeutic monitoring.
- Therapeutic target identification: Investigating modulators of RANKL, activin A, OPN, oxidative stress, and epigenetic/miRNA pathways in organotypic cultures and relevant *in vivo* models.
- Optimized local delivery platforms: Developing biodegradable, sustained-release carriers suitable for the middle-ear environment to minimize ototoxicity and ensure retention.
- Standardization of translational models: Refining organotypic, co-culture, and *in vivo* systems to capture epithelial–stromal–immune–bone interactions, enabling reproducible evaluation of molecular interventions.

Focusing on these priorities provides a framework for mechanism-based, patient-specific adjuncts that complement surgery, reduce recurrence, and mitigate bone destruction. These strategies offer promising avenues for future translational and clinical investigation. Translational implementation must carefully address safety considerations specific to the middle-ear environment. Potential ototoxicity, vestibular effects, mucosal tolerance, and local pharmacokinetics represent important limitations that warrant systematic evaluation. Preclinical models and early-phase clinical studies should therefore incorporate rigorous auditory and vestibular safety endpoints alongside molecular and radiologic measures of efficacy.

## Conclusion

Cholesteatoma is a benign but locally aggressive disease with substantial risk for hearing loss, bone destruction, and recurrent surgery. Surgical excision remains the primary management strategy; however, high recurrence rates and variable outcomes underscore the need for adjunctive, mechanism-driven therapies. Advances in understanding the lesion’s inflammatory milieu, osteoclast activation, biofilm persistence, and molecular regulatory networks provide opportunities for precision interventions delivered directly to the middle ear, with the goals of stabilizing disease, limiting tissue damage, and preserving auditory function.

Future management may integrate surgical excision with locally targeted therapies, informed by molecular and imaging biomarkers to identify high-risk patients and monitor treatment response. Translational studies and early-phase trials focusing on axis-specific interventions, safe and effective local delivery platforms, and robust functional endpoints could help transition cholesteatoma care from reactive surgery to predictive, proactive, and personalized strategies. By aligning mechanistic insights with patient-centered clinical implementation, such approaches have the potential to reduce recurrence, minimize complications, and improve long-term hearing and quality of life for affected individuals.

## Abbreviations

EMT: epithelial-to-mesenchymal transition
IL: interleukin
JAK–STAT: janus kinase/signal transducer and activator of transcription
MAPK: mitogen-activated protein kinase
MMP: matrix metalloproteinase
NF‑κB: nuclear factor kappa B
OPN: osteopontin
PTHrP: parathyroid hormone-related protein
RANKL: receptor activator of nuclear factor kappa‑B ligand
ROS: reactive oxygen species

## Declarations

### Copyright

© The Author(s) 2026.
